# A Novel Method for Effective Closure of Mucosal Defects After Endoscopic Full‐Thickness Resection Using a Dual‐Channel Endoscope

**DOI:** 10.1111/den.15080

**Published:** 2025-07-07

**Authors:** Geng Qin, Guanyu Chen, Shiyu Du

**Affiliations:** ^1^ Department of Gastroenterology China‐Japan Friendship Hospital Beijing China; ^2^ China‐Japan Friendship Hospital (Institute of Clinical Medical Science) Chinese Academy of Medical Sciences and Peking Union Medical College Beijing China

**Keywords:** endoscopic full‐thickness resection, endoscopy, gastrointestinal tumors

## Abstract

Watch a video of this article.

Endoscopic full‐thickness resection (EFTR) has emerged as a preferred therapeutic modality for the treatment of submucosal tumors, including gastrointestinal stromal tumors [1]. Despite its growing use, post‐EFTR closure remains technically challenging due to difficulties in approximating and securing the mucosal edges [2, 3]. These challenges often hinder effective closure and increase the risk of complications.

To overcome these limitations, we have developed a novel closure technique employing a dual‐channel endoscope, designed to facilitate precise and efficient wound approximation. The two working channels of the endoscope (GIF‐2TQ26OM) are designated as Channel A and Channel B, with titanium clips deployed through each referred to as A‐clips and B‐clips, respectively.

During the closure procedure, only a single A‐clip is used throughout. This clip is employed to grasp and retract the mucosa (or mucosa with the muscularis propria) from one side of the defect, aligning it linearly with the opposing edge (Figure 1B). Once proper alignment is achieved, one or more B‐clips are applied to approximate the bilateral mucosal edges and secure the closure (Figure 1C,E). The A‐clip is then released and repositioned to repeat the process on the next section of the defect (Figure 1D). After completing the placement of B‐clips, the A‐clip performs the final approximation to complete the closure. Figure 2 is an illustration.

**FIGURE 1:**
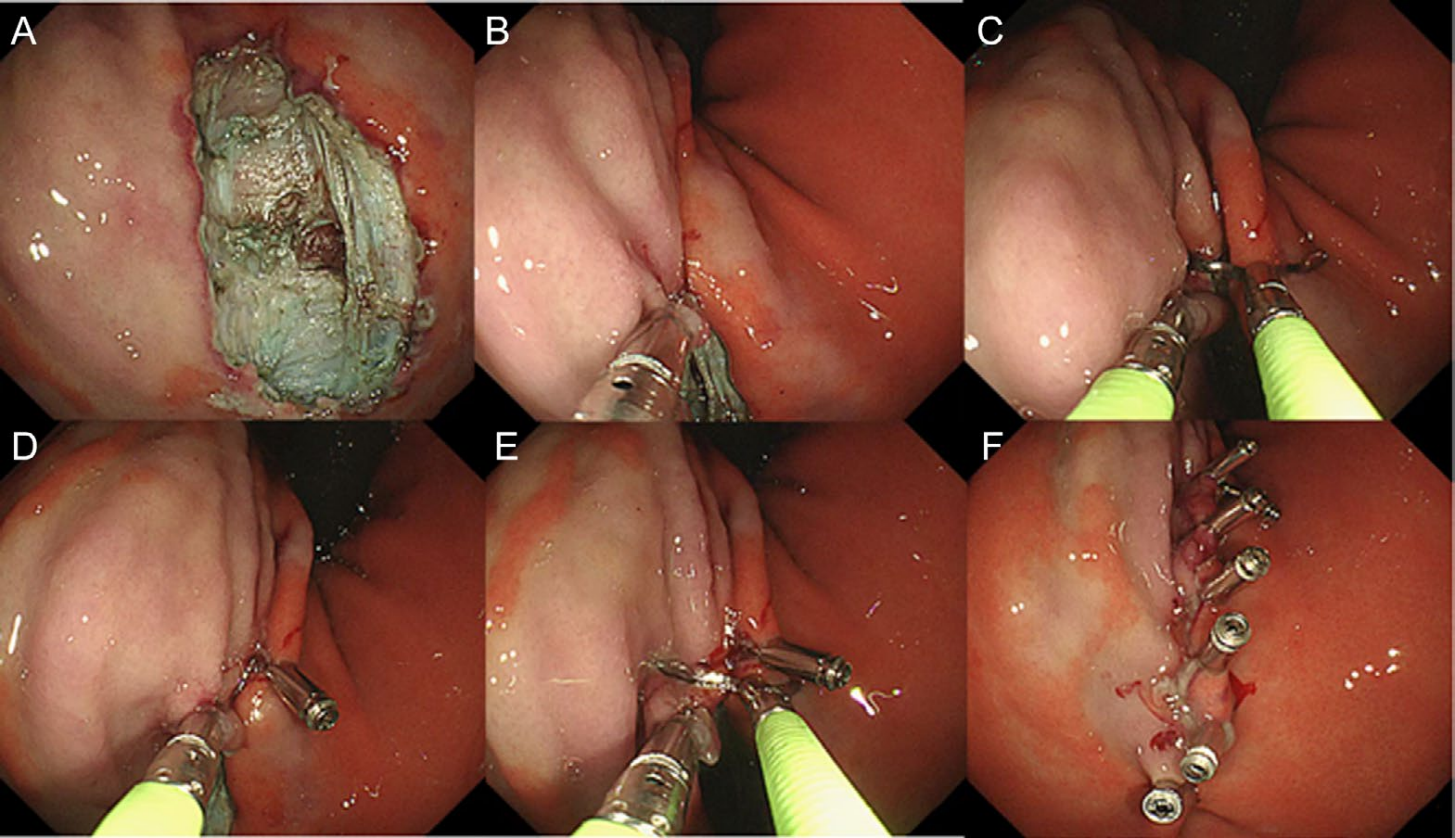
(A) Postoperative wound. (B, D) Placement of an A‐type titanium clip to grasp and retract the mucosa (or mucosa and muscularis) on one side of the wound, facilitating linear alignment with the opposing mucosal edge. (C, E) Deployment of B titanium clips to approximate and secure the aligned mucosal edges. (F) Following closure with all B‐clips, the A titanium clip is utilized to complete the final wound closure.

**FIGURE 2:**
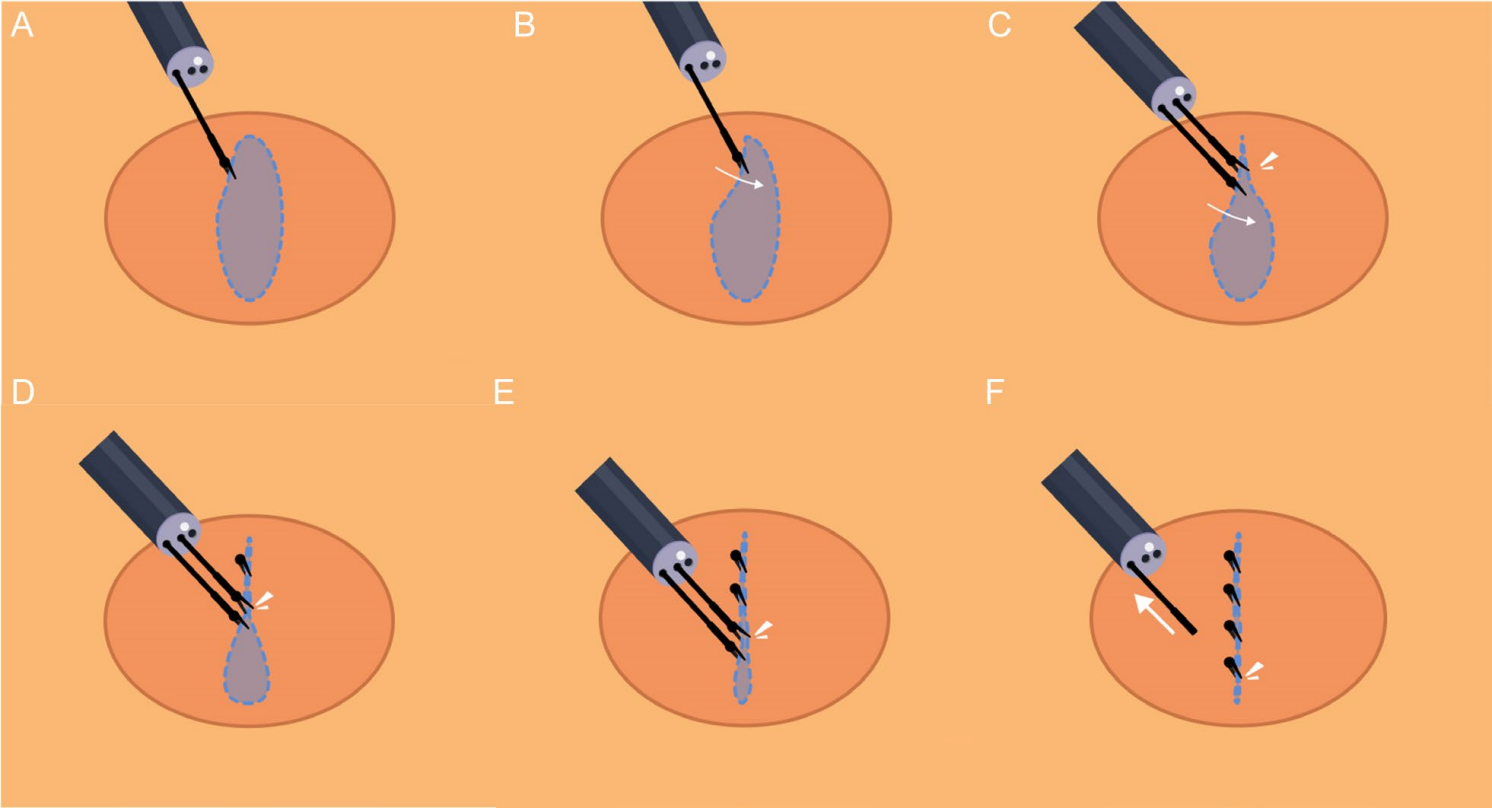
(A) Placement of the A titanium clip to grasp the mucosa (or mucosa and muscularis) on one side of the wound. (B) Using A titanium clip to retract the mucosa and achieve linear alignment with the opposing mucosal edge. (C–E) Deployment of B titanium clips to approximate and secure the aligned mucosal edges. (F) Following closure with all B‐clips, the A titanium clip is utilized to complete the final wound closure.

This technique has been successfully applied in clinical practice, as demonstrated in the accompanying video (Video S1), confirming its feasibility and effectiveness in real‐world EFTR cases.

The dual‐channel endoscopic technique offers multiple advantages: improved mucosal alignment, reduced clip span, shorter procedural time, and enhanced surgical precision. Collectively, these benefits contribute to increased procedural efficiency and potentially lower complication rates.

## Author Contributions

Geng Qin designed and performed the research, collected and analyzed the data. Geng Qin and Shiyu Du offered funding support. Guanyu Chen drafted and revised the manuscript.

## Conflicts of Interest

The authors declare no conflicts of interest.

## Supporting information


**Video S1.** Demonstration of a novel method for effective closure of mucosal defects after endoscopic full‐thickness resection using a double‐channel endoscope.
